# Interferon-Alpha Induced and Ribavirin Induced Thyroid Dysfunction in Patients with Chronic Hepatitis C

**Published:** 2010-06-01

**Authors:** Amina Nadeem, Muhammad Mazhar Hussain, Muhammad Aslam, Tassawar Hussain

**Affiliations:** 1 Department of Physiology, Army Medical College, Rawalpindi, Pakistan; 2 Department of Physiology, Shifa College of Medicine, Islamabad, Pakistan; 3 Hearts International Hospital, Islamabad, Pakistan

**Keywords:** Chronic Hepatitis C, Interferon, Ribavirin, Thyroid Disease

## Abstract

Chronic hepatitis C (CHC) is one of the commonest infectious diseases of the liver and may lead to cirrhosis or hepatocellular carcinoma. Combination therapy with pegylated interferon (PEG-IFN) and Ribavirin is the treatment of choice for CHC. Combination therapy is thought to act by means of antiviral mechanisms and immunomodulation. Thyroid dysfunction is the most common autoimmune adverse effect associated with combination therapy; hypothyroidism is more common than hyperthyroidism. Antithyroid antibodies and female sex have a predictive value in the development of interferon induced thyroid disease (IITD). Patients with CHC should be informed of the possibility of side effects on the thyroid gland. Screening for antithyroid antibodies and thyroid function tests should be performed in patients with CHC before the commencement of antiviral treatment, and during and after it. This article reviews different aspects of IITD, including its pathogenesis, clinical manifestations, association with treatment regimens and treatment response and the outcome of thyroid dysfunction.

## Introduction

**Figure 1:**
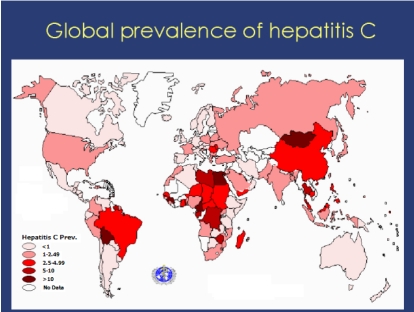
Global prevalence of hepatitis C

Hepatitis C virus (HCV) has been given many names, ‘the silent epidemic’, ‘the silent dragon’ and ‘the disease of the new millennium’. According to studies carried out by the World Health Organization approximately 170 million individuals (3% of the world’s population) have been diagnosed with HCV [1] Fig. 1. Chronic hepatitis C (CHC) is considered to account for about 70 to 75% of all cases of chronic hepatitis and 15-20% of all cases of cirrhosis of the liver and hepatocellular carcinoma [2]. Pegylated interferon (PEG-IFN) is recommended as the standard therapy for chronic hepatitis C. More recently, PEG-IFN has been recommended for the treatment of chronic hepatitis C in a once-daily dosage. Interferon (IFN) -alpha and Ribavirin act by immunomodulation and antiviral mechanisms; they also have direct toxic effects on the thyroid [3]. Production of antithyroid antibodies and thyroid dysfunction are the most common autoimmune disorders associated with combination therapy [3][4][5][6][7][8]. Other extra hepatic diseases  related to IFN therapy in chronic hepatitis C are diabetes mellitus, haematological disease and neuropsychiatric disturbances.

## Antithyroid Autoantibodies in Chronic Hepatitis C Patients

Autoantibodies are commonly found in patients with CHC, even without any treatment Table 1. These include antinuclear antibodies (ANA), anti smooth muscle antibodies (ASMA), liver/ kidney antibodies, antimicrosomal antibodies (AMA), thyroperoxidase antibodies (TPO) and antithyroglobulin antibodies (TGA) [5]. The overall frequency of non-organ-specific antibodies is found to be significantly higher in anti-HCV positive patients than in normal healthy individuals [4]. These autoantibodies persist in patients with CHC; and are detectable even after treatment [7]. It has been well-documented by various studies that treatment of chronic hepatitis C infection with IFN-alpha and Ribavirin combination-therapy can lead to the induction of thyroid autoantibodies [6][7][8][9] Table 2. The presence of AMA or antithyroperoxidase (anti-TPO) antibodies before antiviral treatment is a strong predictive factor of IFN-induced thyroid dysfunction [8]. Recent data suggest that females carry a higher risk of anti-thyroid autoantibodies than male patients, and the risk in females increases directly with increasing age [6].

Thyroid autoimmunity has been detected in patients with chronic hepatitis C, even in the absence of IFN treatment, cirrhosis, hepatocellular carcinoma, or in normal controls [4]. Treatment either with IFN alone or with combination IFN and Ribavirin therapy carries an equal risk of the development of thyroid antibodies (TGA and TPO Abs) [10]. The presence of thyroid autoantibodies before IFN treatment increases the risk of a rise in the autoantibody titer during or after IFN treatment. IFN can induce or aggravate autoimmunity in CHC patients [10]. Response to treatment is also not related to the presence or the percentage of thyroid autoimmunity, as the incidence in long term responders is not significantly different than in non-responders. Moreover; progressive deterioration in liver disease also has no relationship to the occurrence of thyroid autoimmunity during IFN therapy [11]. The appearance of thyroid autoantibodies during treatment with IFN, in most cases, is related to the occurrence of a destructive process in the thyroid gland. Recently it has been reported that the clinical outcome of thyroid disease in these patients is correlated to the viral load, the continuation of antiviral therapy, the genotype of the virus, the age and gender of the patients. Antithyroid autoantibodies appear in both treated and untreated patients, which suggests that autoimmunity is linked to HCV infection itself and not to antiviral therapy alone [12]. It has been suggested that HCV induces the endogenous IFN -alpha and -beta in the thyroid gland, as a part of innate immune response to viral infection. Endogenous and exogenous IFN lead to the activation of natural killer (NK) cells, memory T cells, dendritic cells; it also causes the production of thyroid antibodies [13]. The antithyroid antibodies may persist in CHC patients after cessation of IFN therapy. The incidence of autoantibodies in CHC patients with or without IFN treatment is reported to be 10-45 % [4],[9][10][11].

**Table1:** Frequency of autoantibodies in chronic hepatitis C patients without IFN therapy

**Year**	**Antibodies^a^**	**CHC Patients**	**Controls**	**P-value**	**Reference**
1999	ANA	42.1%	NR	NR	47
2004	TGA	17%	NR	NR	5
	TPO	21%	NR	NR	
2006	ANA	11.5%	1.9%	0.05	45
	ASMA	13.5%	1.9%	0.027	

^a^ ANA: antinuclear antibodies; TGA: thyroglobulin antibodies; TPO: thyroid peroxidase antibodies; ASMA: anti smooth muscle antibodies; NR: Not reported; CHC: chronic hepatitis C.

**Table2:** Frequency of autoantibodies in chronic hepatitis C patients with IFN therapy.

**Year**	**Antibodies^a^**	**Percentage**	**Reference**
2001	TGO and TPO	47%	11
2001	TGO and TPO	31.5%	44
2001	TGO and TPO	21.1%	37
2002	TGO and TPO	23.6%	10
2002	TGO and TPO	27.7%	9
2006	TGO and TPO	11.6%	18
2009	TPO	24.1%	48

^a^ TGA: thyroglobulin antibodies; TPO: thyroid peroxidase antibodies.

## Interferon-Induced Thyroid Disease (IITD)

The side effects of IFN therapy on the thyroid gland were reported for the first time about two decades ago, and since then numerous studies have been done in this area. Mean TSH levels are found to be higher, and free T3 and free T4 levels are found to be lower, in patients with chronic hepatitis C, showing a tendency to hypothyroidism, even without antiviral treatment [9]. However it has been well-documented that treatment of chronic hepatitis C infection with IFN-alpha and Ribavirin combination therapy can predispose to IITD. The IFNs are a group of cytokines produced by white blood cells, fibroblasts, or T cells produced in response to a viral infection as a part of innate immunity. The incidence of IITD varies widely in different studies, and has been reported to be in the range of 3.9%-27.2% % [3][4][8][11][14][15][16][17][18][19][20][21][22][23]. Meta-analysis of the data reveals a mean incidence of 11.02 % Table3. Substantial research work has been carried out to determine the relationship of thyroid dysfunction and IFN therapy,especially in relation to the production of autoantibodies in these patients in order to determine the association of thyroid dysfunction with the severity of the disease and treatment response and to evaluate predictive factors.

**Table3:** Frequency of IFN-Induced Thyroid Disease (IITD)^a^

**YEAR**	**Total Patients (No.)**	**Positive Cases (No.)**	**Frequency of IITD**	**Reference**
2001	53	6	11.8%	10
2001	136	36	27.2%	44
2002	254	30	11.8%	29
2004	225	24	10.7%	17
2005	221	15	7.0%	15
2005	439	17	3.9%	14
2006	461	58	12.6%	16
2006	95	14	14.7%	18
2009	107	20	18.69%	22
2009	58	06	10.3%	48

^a^ Meta-analysis: Mean Incidence: 11.02%

## Manifestations of IITD

The incidence of IITD varies widely in different studies, and has been reported to be in the range of 3.9%-27.2% % with a mean incidence of 11.02% [3][4][8][11][14][15][16][17][18][19][20][21][22]. This wide range may be due to the fact that in some studies, cases with clinical manifestations were labeled as positive cases, whereas in other studies, subclinical cases were also included in the positive cases, raising the incidence. Moreover, in a few studies, positive cases were determined only over six months of antiviral therapy while in others, the follow-up period was extended up to 6-12 months, increasing the incidence of positive cases. In our study [22], the incidence of those patients developing thyroid dysfunction was 18.69%. Females were at higher risk, and hypothyroidism was more common than hyperthyroidism. The frequency of hypothyroidism was 8.4%; while hyperthyroidism developed in 7.5% of patients. 2.8% of patients showed manifestations of biphasic thyroiditis i.e. transient hyperthyroidism, at 12 weeks of therapy, followed  by hypothyroidism at the end of 24 weeks of treatment.

IITD can manifest both as clinical autoimmune thyroiditis (i.e. Hashimoto’s thyroiditis and Graves’ disease) and as non-autoimmune thyroiditis (i.e. destructive thyroiditis and non-autoimmune hypothyroidism), the latter being a reported 50% of the cases of IITD [3][6][24]. Among IITDs, hypothyroidism is the most common side effect [10][11][20][23][25][26]. Thyrotoxicosis, however, is less frequently observed [5]. Patients who develop symptomatic Graves’ disease with positive thyroid-stimulating antibodies may have persistent IITD, requiring long-term treatment for thyroid dysfunction [5]. IFN therapy may cause two different manifestations of thyrotoxicosis: Graves’ disease or destructive or biphasic thyroiditis [5]. In biphasic thyroiditis, patients can have transient thyrotoxicosis due to the release of preformed thyroid hormones caused by an inflammatory process, which resolves, and then progresses to hypothyroidism [9][27]. These patients do not have antithyroid antibodies and show a low uptake of radioactive iodine in a thyroid scan. Destructive thyroiditis is self-limiting, and permanent hypothyroidism is rare [28]. Patients who develop Graves’ disease have positive thyroid-stimulating antibodies in their blood [5]. Overt hyperthyroidism due to subacute thyroiditis, followed by Graves’ disease, has been reported in the same patient on IFN therapy over a short period of ten months [29]. In France, three patients were reported to have developed Hashimoto’s thyroiditis followed by Graves’ disease over a period of a few months [28]. Braga et al. reported a case in which a female patient, already diagnosed as having Graves’ disease, suffered from CHC infection and developed IFN-induced severe hypothyroidism. Her hyperthyroidism resumed after discontinuation of IFN therapy. It is found that females carry a higher risk of developing IITD [4][7][22][24][30][31]. Although rare, IFN-induced Graves’ opthalmopathy has also been reported [32]. Other predisposing factors include the presence of thyroid autoantibodies and Asian ethnicity [32]. Vertical transmission of HCV infection is well documented in the literature. In a study, 11.1% of untreated chronic hepatitis C children infected through vertical transmission revealed subclinical hypothyroidism, without any evidence of autoantibodies [33]. In addition to clinical manifestations and biochemical changes, IITD also leads to thyroid hypoechogenicity on ultrasonography, which suggests relevant morphological changes in the thyroid gland as well as functional changes [16][34].

## Pathogenesis of IITD

The exact mechanisms in the pathogenesis of IITD in patients with HCV infection are not fully known, but usually these disorders are reversible and only require supervision and treatment, if symptomatic. IITD is considered to be caused by immunomodulation and is associated with the production of autoantibodies [9]. Autoantibodies are not invariably present in all patients developing IITD, which suggests that mechanisms other than autoimmunity can be involved as well [18]. Ribavirin also has immunomodulatory effects and acts alone or synergistically with IFN- alpha to cause IITD [14]. Ribavirin is a guanosine analog and acts by competitively inhibiting inosine monophosphate dehydrogenase, viral RNA polymerase and viral messenger RNA (mRNA) guanylyltransferase, resulting in decreased viral mRNA and protein synthesis. Ribavirin is also incorporated into the viral genome and causes mutation, resulting in a decrease in specific viral infectivity. However, it can also enhance non-virus-induced immune responses and can result in autoimmune diseases in genetically predisposed patients [35]. Ribavirin has a predominantly immunological effect on Th1 cytokines activating CD-8+ T lymphocytes, which induce cellular destruction via cellular immunity [35].

The IFNs belong to a group of proteins called cytokines which are produced by white blood cells, fibroblasts and T lymphocytes as a part of innate immunity in response to viral infection. There are 3 types; alpha, beta and gamma IFN. IFN-alpha is used in the treatment of CHC infection. Recent data suggests that IFN-alpha has direct toxic effects on the thyroid gland, in addition to having immunomodulatory mechanisms [3]. The evidence of direct toxic effects of IFN on the thyroid is derived from the findings of the up-regulation of TSH receptors, increased thyroglobulin, thyroid peroxidase enzymes and sodium iodide symporter protein expression. Moreover, IFN causes induction of heat shock proteins and thyroid cell apoptosis [28]. IFN can also activate natural killer cells, the maturation and proliferation of dendritic and memory T cells, and the prevention of T cell apoptosis, causing a rise in the thyroid autoantibody titer. These antibodies will result in thyroid gland damage in genetically predisposed individuals [35]. Moreover, the human leukocyte antigen-A2 (HLA-A2) is also associated with IFN-alpha therapy-induced autoimmune thyroid dysfunction in patients with chronic hepatitis C [36]. In one study, it was found that aggravation of fibrosis has no association with the occurrence of thyroid autoimmunity and thyroid dysfunction [18], whereas another study showed that low fibrosis is significantly associated with an increased incidence of IITD [17].

It has been suggested that only predisposed patients with CHC develop IITD. This predisposition may be genetic or environmental in origin. In genetically susceptible individuals, IFN-alpha is one of the environmental factors which cause thyroid dysfunction [9]. Although high endogenous IFN-alpha levels may also be associated with naturally occurring autoimmune thyroid disease, HCV itself plays a role in the disease because thyroid disease is reported in CHC patients even without treatment [24]. Moreover, patients with chronic hepatitis B infection who are treated with higher doses of IFN alpha (5 million units as compared to 3 million units in CHC) are less likely to develop thyroid disease than those with chronic HCV infection [13]. This indicates that HCV and IFN-alpha may have a synergistic role in the pathogenesis of IFN-induced thyroid disease. HCV causes the production of endogenous IFN-alpha and -beta in the thyroid gland as part of innate immunity. It is found that HCV envelope protein (E2) can bind to CD 81 molecules in thyroid cells, inducing IL 8 secretion and activating resident T cells in genetically susceptible individuals [28]. It also suggests that IITD is not associated with dose-response relationship [13]. In various studies, different clinical pictures of IFN-induced thyroid disease occur in CHC patients including hypothyroidism, hyperthyroidism or biphasic thyroiditis. Moreover, different manifestations of thyroid disease can occur in the same patient during or after treatment, suggesting a possible change in the immunological process induced by IFN treatment in genetically susceptible individuals. Overt hyperthyroidism due to subacute destructive thyroiditis, followed by Graves’ disease, has been reported in the same patient on INF therapy over a short period of ten months [29].

The patients who develop Graves, type thyrotoxicosis induced by IFN therapy show a different immunological response from those showing hypothyroidism or transient thyrotoxicosis caused by a destructive process in the thyroid gland. Type 1 immune response plays a critical role in the development of many autoimmune diseases and in the resolution of viral infection. In thyroid autoimmune diseases, both T helper (Th) cells 1 and Th 2 responses coexists in the same patient with CHC [37]. In Hashimoto’ s thyroiditis and in silent thyroiditis in otherwise normal individuals i.e. not suffering from CHC, type 1 immune response depicting cellular immunity is enhanced, as demonstrated by an increase in serum Th 1 cytokines leading to hypothyroidism [37]. In Graves’ disease, type 2 immune response, depicting humoral immunity by the activation of Th cells 2, is associated with an increase in thyroid-stimulating autoantibodies to thyrotropin receptors [37]. It is speculated that the same immune mechanisms may be operating in the development of different immunologic responses in IITD [12]. IFN causes intracellular signaling through the JAK-STAT pathway, and induces the expression of cytokine and adhesion molecule genes. IFN increases the expression of the MHC-I antigen on thyroid epithelial cells, activating cytotoxic T cells. The result is inflammation leading to tissue damage [28]. Although response to IFN therapy is related to both host and viral factors, genetic background seems to play a predominant role in determining thyroid autoimmunity with IFN [38]. Only in those subjects genetically predisposed to developing autoimmune disease, can combined IFN plus Ribavirin treatment trigger a Th1-mediated cytotoxic process in the thyroid gland [32]. Why IFN causes Graves’ disease in CHC patients is not fully known as Graves’ disease is caused by TH-2 mediated humoral immunity which has not been reported to be induced by IFN. One possible explanation could be the probability that the initial phase of Graves’ disease is Th-1 mediated [28]. Presence of antithyroid antibodies, female gender and Asian ethnicity being the independent predictors of IITD indicate the role of genetic susceptibility. Susceptible genes have been identified, including HLA-DR, CTLA and PTPN 22, thyroglobulin and TSH receptor genes [28]. Moreover, some evidence of the association of IITD with CTLA-4 and CD-40 gene polymorphism has been found [28]. PEG-IFN plus Ribavirin have been reported to be associated with the occurrence of central hypothyroidism and hypophysitis in CHC patients, which suggests a possible central role [29].

## Association of IITD with IFN Treatment Regimens

There are varying results reported in the literature regarding the association of IITD with treatment regimens in patients with CHC. Patients treated with a combination IFN and Ribavirin therapy are reported to be more susceptible to IITD than those treated with IFN alone [6]. In another study, the addition of Ribavirin to IFN therapy did not increase the risk of thyroid dysfunction compared to IFN alone [4]. Carella C et al. [10] showed that IFN and Ribavirin combined, as compared to IFN alone, did not have a significant difference in the occurrence of thyroid autoantibody patterns, but the former group of patients had a higher risk of hypothyroidism. However, very few studies investigated the role of Ribavirin in autoimmune diseases, specially thyroid autoimmune disorders. Ribavirin has been used in combination with IFN in CHC patients and never alone, so the exact contribution of Ribavirin itself in the pathogenesis of IITD in CHC can not be fully comprehended.

Regarding those patients who undergo two therapeutic courses, the patients treated for the second time with IFN and Ribavirin were protected from the development of thyroid autoimmunity and/or thyroid dysfunction, (unlike those who were treated with IFN alone, as a 1st therapeutic regimen, and were negative for thyroid autoantibodies) [6]. The incidence of thyroid dysfunction is similar for standard IFN and PEG-IFN treated patients, so PEG-IFN is not a risk factor, compared to standard IFN [24]. There are varying results in percentage of thyroid dysfunction from the first to the second therapeutic schedule of IFN. Some studies reveal that in patients undergoing two consecutive antiviral treatments, the frequency of hypothyroidism increases significantly from the first to the second therapeutic schedule, [39] while in others there is no significant difference in the frequency of thyroid dysfunction during the first and the second treatments [5]. Further studies and long-term follow-up of patients undergoing two consecutive antiviral courses can help to draw a definite conclusion.

## Association of IITD with Response to IFN Treatment

The association of the development of IITD with long term remission of CHC needs to be investigated further. There are varying results regarding the association of IITD and response to treatment. In some studies, the development of hypothyroidism in patients with thyroid autoantibodies who undergo treatment with IFN-alpha plus Ribavirin is significantly associated with better treatment response in CHC patients [39][40][41]. One study revealed that the recovery rate from CHC is better in non- autoimmune-type thyroid disease, without any autoantibodies in the serum, than in controls in the same cohort [31]. Other studies show that thyroid dysfunction during the treatment of chronic hepatitis C with IFN-alpha has no association with efficacy of therapy [42]. Whether the immune mechanisms involved in the pathogenesis of thyroid disorders induced by IFN are the same as those regulating the therapeutic response in CHC patients is not yet clear. The percentage of thyroid autoimmunity and thyroid dysfunction in long-term responders is not significantly different than that in non-responders. Response to therapy is dependent on many factors including viral load, virus genotype, adherence to therapy, degree of fibrosis in the liver, and is not dependent on the absence or presence of IITD. Therefore, thyroid autoimmunity and dysfunction induced by IFN-alpha therapy cannot be considered as predictors of treatment response or as valid prognostic markers of the progression of liver disease [8].

## Clinical Features, Diagnosis and Treatment of IITD

Common symptoms of hypothyroidism include easy fatigability, anorexia, body aches and depression; whereas nervousness, irritability, increased appetite, fatigue, insomnia, and weight loss are common in overt hyperthyroidism. These symptoms can easily be attributed to HCV infection itself. Thyroid dysfunction due to non-specific symptoms can easily remain undiagnosed, if patients do not undergo periodic screening for thyroid dysfunction. The best way is to monitor the TSH levels of all patients with CHC undergoing treatment before, during and after IFN therapy. Periodic serum levels of T3, T4, TSH and antithyroid antibodies during IFN therapy and in the follow-up period should be closely monitored for all patients found to be positive for IITD. Thyroid scans should be done if indicated.

The diagnosis of Hashimoto’s thyroiditis is based on hypothyroidism with positive thyroid antibodies (anti-TPO, anti-TG). Destructive thyroiditis is defined by transient thyrotoxicosis with reduced radioiodine uptake and negative thyroid antibodies, ,followed by a hypothyroid phase. The diagnostic citeria for Graves’ disease is a thyrotoxicosis with either positive TRAb (TSH receptor antibodies) and/or a diffusely increased thyroid scan uptake [32].

Treatment for hyperthyroidism is usually symptomatic. Beta blockers are given for it. Glucocorticoids are contraindicated in CHC patients. In severe forms of Graves’ disease, surgery or radioactive ablation may be indicated if the Graves’ disease persists even on cessation or completion of IFN therapy.

Patients who are taking treatment for thyroid disease prior to IFN therapy may require increased doses of thyroid medication during antiviral treatment.Doses then have to be decreased on completion of IFN therapy. Patients who develop IFN-induced symptomatic hypothyroidism are treated with oral thyroxine and usually do not require a decrease in the IFN dose or the cessation of antiviral therapy. They may require increased doses of thyroxine if they are given a second course of IFN [28]. Hypothyroidism in most patients is usually reversible on completion of antiviral treatment.

## Outcome of IITD

There are limited studies available on long-term follow-up of CHC patients with IITD. Most of the data available is about IITD during, at the end of IFN treatment and six months after it. . IFN therapy is not contraindicated in the presence of autoantibodies , as most patients with increased antibody titer recover on completing therapy. In most patients IITD is reversible, usually on cessation of antiviral therapy [12][33]. All patients with IFN-alpha-induced thyroid disorders may not require any treatment for IITD, or may be controlled with medication for thyroid disease and be able to continue antiviral therapy. In some cases, though, reduction in the dose of IFN and Ribavirin, and in some others, even cessation of antiviral therapy may be necessary, especially in thyrotoxicosis patients [3][40]. Which factors determine the reversibility of IITD on completion of IFN therapy are yet to be determined by further research in this field. The reversibility of IITD in comparison to its irreversibility was not found to associated with the age of the patient, the HCV genotype, the virus type of IFN, the timing of the onset of IITD, the cessation of IFN therapy and thyroid hormone levels [40]. Nevertheless, the IFN-alpha-induced thyroid disorder is not always reversible and may require life-long treatment. Not only should the development of autoimmune thyroid disorders during IFN-alpha therapy be watched, but also the outcome of the thyroid disease should be followed up [40]. Long-term hypothyroidism may persist in a small group of patients (approximately 2%) and may require the lifelong use of thyroid replacement therapy [33][42]. Thyroid function tests should be checked prior to therapy, periodically during therapy, and at least once in the six months following therapy. A longer follow-up may be needed in positive cases in order to confidently establish the complete resolution of thyroid disease [14].

The disease is not reversible in all cases of IFN-alpha-induced autoimmune thyroid dysfunction. Therefore, it is necessary that autoantibodies and thyroid function be monitored during therapy, and the course of thyroid disease be observed if autoimmune thyroid disorder develops during IFN-alpha and ribavirin treatment [43][44][45][46].

## Conclusions

IITD is a common complication of IFN therapy in chronic hepatitis C patients. IFN can induce different manifestations of thyroid disease, including clinical autoimmune thyroiditis (i.e. Hashimoto’s thyroiditis and Graves’ disease) and non-autoimmune thyroiditis (i.e. destructive thyroiditis and non autoimmune hypothyroidism) in genetically susceptible individuals. Hypothyroidism is the most common manifestation. Female sex, Asian ethnicity and the presence of antithyroid antibodies are the independent predictors of IITD. HCV itself plays a synergistic role in the pathogenesis of IFN-induced thyroid disease. Most patients can continue IFN therapy despite the occurrence of IITD, although a few patients may require a reduction in dose or cessation of IFN therapy due to severe manifestations. Chronic hepatitis C patients undergoing IFN therapy should be regularly monitored before the onset, during and after treatment for the development of autoantibodies and thyroid dysfunction.

## Future Studies

IITD needs to be explored further. The exact pathogenesis of IITD is not yet clear. What is yet to be determined is the extent of IFN-induced immunomodulation in patients which can result in such diverse clinical manifestations. A few questions still remain unanswered. If genetic susceptibility determines the occurrence of IITD, why does IITD not occur in chronic hepatitis B patients who are given higher doses of IFN? Although HCV E2 proteins affect the thyroid cells, what is the exact contribution/synergistic effect of CHC infection itself in the pathogenesis of IITD? What is the exact pathogenesis of thyroid dysfunction occurring in some and not in all CHC patients, even without IFN therapy? What factors determine whether or not a patient will develop reversible thyroid disease? What is the association of IITD with treatment response? Long-term follow-up of IITD patients is scarce in the literature. Areas of future research are long-term follow-up of positive cases of IITD to determine reversible/irreversible thyroid dysfunction and its relation to sustained virological treatment response and relapse. Long-term follow-up of negative cases of IITD should be carried out to determine late development of thyroid dysfunction, if any.

Reported cases of chronic hepatitis C worldwide are just the tip of the iceberg. Extensive studies are required in this area to understand the pathogenesis of the disease and treatment-related side effects. Future areas of research are its immunological basis, and the role of genetic, viral and environmental factors, in the etiology of IITD. Better knowledge of these aspects can lead to a better understanding of the pathogenesis of the disease, to improvement in the management of patients undergoing IFN therapy, to earlier diagnosis of the thyroid side effects and to a more appropriate therapeutic approach to IITD.
